# Transmission Dynamics of African Swine Fever Virus, South Korea, 2019

**DOI:** 10.3201/eid2707.204230

**Published:** 2021-07

**Authors:** Dae Sung Yoo, Younjung Kim, Eune Sub Lee, Jun Sik Lim, Seong Keun Hong, Il Seob Lee, Chung Sik Jung, Ha Chung Yoon, Sung Hwan Wee, Dirk U. Pfeiffer, Guillaume Fournié

**Affiliations:** Animal and Plant Quarantine Agency, Gimcheon, South Korea (D.S. Yoo, E.S. Lee, S.K. Hong, I.S. Lee, C.S. Jung, H.C. Yoon, S.H. Wee);; Jockey Club College of Veterinary Medicine and Life Sciences, City University of Hong Kong, Hong Kong, China (Y. Kim, D.U. Pfeiffer);; Kangwon National University College of Veterinary Medicine, Chuncheon, South Korea (J.S. Lim);; Royal Veterinary College, London, UK (D.U. Pfeiffer, G. Fournié)

**Keywords:** African swine fever, transmission dynamics, mathematical modeling, disease outbreak, infectious disease epidemiology, contact tracing, viruses, South Korea

## Abstract

African swine fever (ASF) is a substantial concern for global food production and security. However, lack of epidemiologic data in affected areas has limited the knowledge of the main drivers of ASF virus (ASFV) transmission. To assess the role of vehicle movements and wild boar populations in spreading ASFV to pig farms in South Korea, we combined data generated by ASF surveillance on pig farms and of wild boars with nationwide global positioning system–based tracking data for vehicles involved in farming activities. Vehicle movements from infected premises were associated with a higher probability of ASFV incursion into a farm than was geographic proximity to ASFV-infected wild boar populations. Although ASFV can spill over from infected wild boars into domestic pigs, vehicles played a substantial role in spreading infection between farms, despite rapid on-farm detection and culling. This finding highlights the need for interventions targeting farm-to-farm and wildlife-to-farm interfaces.

African swine fever (ASF) is a highly contagious hemorrhagic viral disease that affects domestic pigs and wild boars. Since its introduction into China in 2018 (*1*) and subsequently into many other countries in Asia (*2*), most of the global pig population has been exposed to the ASF virus (ASFV). In the absence of vaccines and treatments, ASF control relies heavily on on-farm biosecurity and on early detection and containment of infected premises (IPs). It is, therefore, essential to identify and target major ASFV transmission routes. However, only a few studies have assessed the contribution of different transmission routes to ASF epidemics (*3*–*6*), probably because detailed epidemiologic data are lacking. Although those studies have contributed to knowledge of risk factors for ASFV infection, their findings are limited by possible bias resulting from underreporting of outbreaks, absence of information about contact patterns between farms, or both.

After ASFV is introduced into domestic pigs, contact between farms may contribute greatly to virus spread (*7*). Vehicles connect farms through the movements of animals, persons, feed, or medical supplies. Such vehicle movements may create conditions for large ASF epidemics on pig farms, as has been reported for other animal diseases (*8*–*10*). However, despite their probable epidemiologic role, the role of vehicle movements in shaping ASF epidemics has not been assessed. Moreover, although the role of livestock movements in the dynamics of several animal diseases has been assessed in a large body of modeling studies (*11*,*12*), other types of contact between farms, such as those mediated by vehicles involved in farming activities, have rarely been explicitly accounted for.

In 2019, South Korea experienced its first ASF outbreak, which affected domestic pigs and wild boars in the northernmost part of the country. At least 1 pig was positive for ASFV by reverse transcription PCR (*13*) on 14 farms (IPs) from September 17 through October 9. ASFV infection was also confirmed by reverse transcription PCR for 26 wild boars from October 3 through November 20. We assessed the contribution of vehicle movements and wild boars to the spread of ASFV to pig farms during the 2019 epidemic in South Korea by combining ASF case data and vehicle movement data generated by nationwide global positioning system (GPS) tracking.

## Methods

### Data

**Figure 1:**
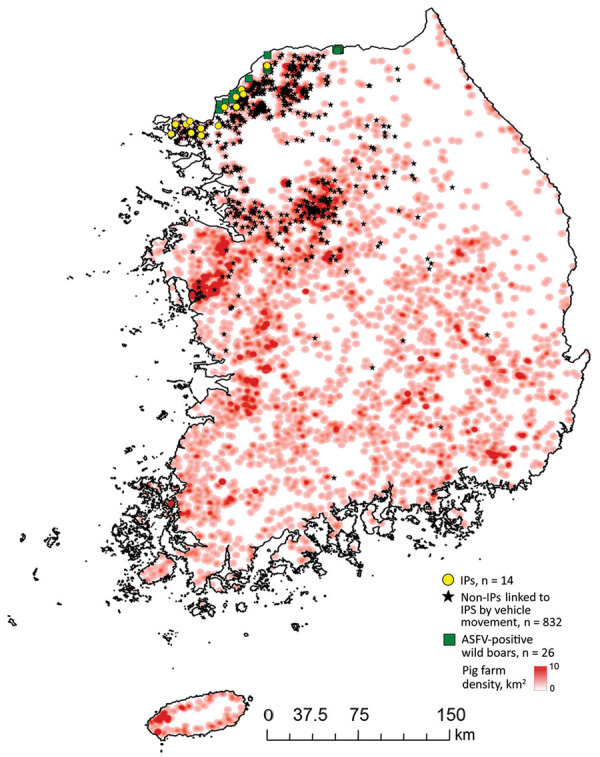
Spatial distribution of registered domestic pig farms in South Korea, indicating African swine fever–positive farms (IPs); ASFV-positive wild boars, confirmed during the study period (August 28–October 16, 2019); and pig farms visited by vehicles that had visited IPs >1 time during the study period. ASFV, African swine fever virus; IP, infected premises.

**Figure 2:**
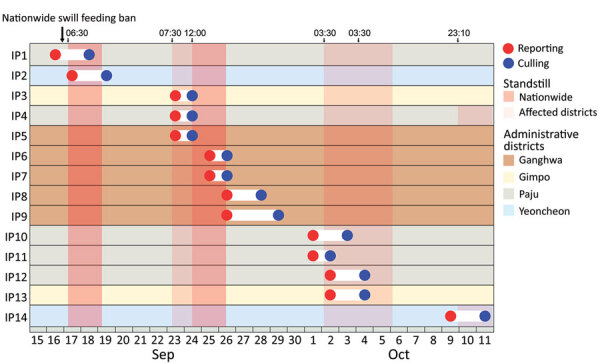
Timeline of reporting and culling of African swine fever virus IPs and control measures implemented during the African swine fever epidemic in South Korea, 2019. Reddish vertical shades represent movement restriction (standstill) imposed across the country (darker shades) or only in the affected municipalities (lighter shades). Numbers on the top represent the time when movement restriction was imposed. The colors of horizontal shades refer to IPs’ municipalities. IPs were numbered in the order of reporting dates. Over the course of the epidemic, six 48-hour standstill periods (bans on movements of livestock, persons, vehicles, and supplies to farms and slaughterhouses) were enforced across the country or in affected municipalities. IP, infected premises**.**

**Figure 3:**
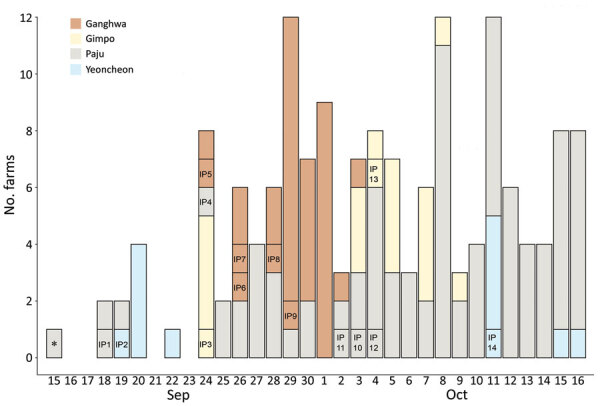
Number of farms registered in the government pig farm database that were emptied during the study period by culling or government purchase, South Korea, 2019. IP labels are shown on the day they were culled. Herds located within a 3-km radius around IPs and herds with an epidemiologic link to IPs (e.g., same ownership) were culled within 3 days after confirmation of African swine fever virus infection in IPs. As the epidemic developed, the government further depopulated remaining herds in affected municipalities as a preventive measure. *One farm stopped rearing pigs for reasons not associated with the epidemic. IP, infected premises.

The Animal Plant and Quarantine Agency (https://www.qia.go.kr) provided the domestic pig farm registry and farm case data. The study population included all 6,340 registered pig farms (Figure 1). IPs were in 4 contiguous municipalities: Ganghwa Island (n = 5/35), Gimpo (n = 2/20), Paju (n = 5/93), and Yeoncheon (n = 2/80) (*13*). Any 2 IPs were <84 km apart (median 28.5 km) (Appendix Figure 1). By October 16 (i.e., 1 week after the last reported IP), 62.7% (143/228) herds in affected municipalities had been depopulated (Figures 2,3).

Most (71.4%) IPs raised >1,000 pigs (Appendix Figure 2). Premises were either commercial (n = 12) or backyard (n = 2) farms: 10 breeding and fattening, 2 breeding, and 2 fattening farms. All IPs, except for 1 backyard farm, were registered. Of the 14 IPs, 11 were detected through farmers’ reports of ASF-like clinical signs and 3 were detected by active surveillance. At the time of reporting, <5 pigs on each farm showed ASF-like clinical signs; these clinical signs were observed in ASFV-positive sows on 9 IPs.

Data on the movements of GPS-tracked vehicles involved in farming activities (e.g., private and government veterinary services; feed, manure, and livestock transport) were collated from the Korean Animal Health Information System (https://www.kahis.go.kr). Given the low number of symptomatic pigs at the time of reporting and the estimated incubation period in pigs (4–13 days) (*14*), we assumed that the length of time between farm infection and reporting was <20 days. In addition, because the law required that vehicles be disinfected before entering farms and when entering and exiting a city, town, or village, we assumed that ASFV-contaminated vehicles remained infectious for <1 week. On the basis of these assumptions, we considered all movements made by vehicles that entered IPs from August 28 (20 days before the first report of an infected premise) through October 16 (a week after the last report of an infected premise).

The Ministry of Environment (https://me.go.kr) provided data on cases in wild boars. From the first report of ASFV infection in domestic pigs, surveillance efforts for wild boars progressively increased by providing financial incentives for wild boar hunting, trapping, and carcass reporting and by testing for ASFV all wild boars caught or found dead (Appendix Figure 3). We assessed spatial clustering of wild boar cases by using an elliptic version of the spatial scan statistic in SatScan (https://www.satscan.org).

### Bayesian Modeling 

To test the hypothesis that vehicle movements and ASFV-infected wild boars were the main sources of infection for pig farms, we fit a model of ASFV transmission to the farm case data. A vehicle was considered potentially contaminating if it entered farm *i* within *d* days after having visited farm *j* while farm *j* was infectious. For a given farm on a given day, the overall force of infection was modeled as the sum of the risk for infection resulting from visits by potentially contaminating vehicles, the risk resulting from exposure to wild boars in the spatial clusters of ASFV-positive wild boars, and background risk. Two levels of background risk were considered, depending on the location of a farm: in municipalities where the virus had been detected or across the country. We estimated parameters by using a 2-stage Metropolis-Hastings Markov chain Monte Carlo algorithm and, because infection dates were not observed, a data augmentation technique (*15*–*17*). The model accounting for the influence of vehicle movements and wild boars was compared with models accounting for only 1 of these epidemiologic factors or for only the constant background risk (null model) on the basis of their deviance information criterion (Appendix).

## Results

### Vehicle Movement Patterns

**Figure 4:**
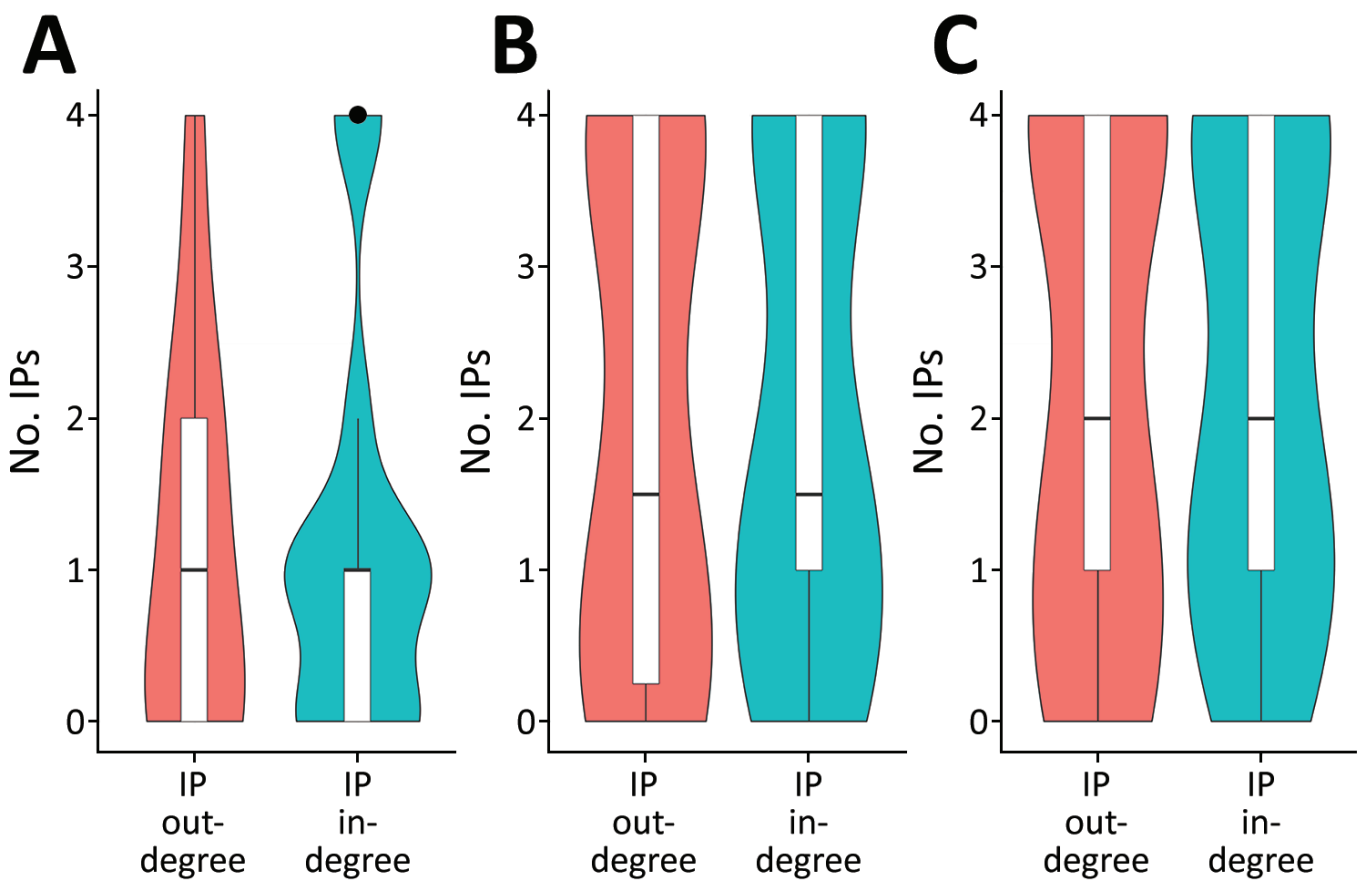
Distribution of the number of African swine fever virus–infected premises connections through vehicle movements, South Korea, 2019. IP out-degree represents the number of other IPs to which an IP sent >1 vehicle; IP in-degree represents the number of other IPs from which an IP received >1 vehicle. With 1-day (A), 3-day (B), or 6-day (C) assumptions for the duration of vehicle infectiousness, only the movements made up to 20 days before an exit farm reported suspicion of ASFV infection and before an entry farm reported suspicion of infection were considered. In the boxplots, center horizontal lines represent medians, and box limits represent upper and lower quartiles. Upper and lower whiskers extend to the largest and smallest values within 1.5× interquartile ranges. The point represents an outlier. ASFV, African swine fever virus; IP, infected premises.

During the study period, 208 vehicles visited IPs, making 12,671 visits to 832 farms (infected and noninfected). A total of 156 vehicles made 2,824 farm visits within 3 days after having visited an IP (assuming that vehicles could remain contaminated for 3 days after visiting an IP); each vehicle made a median of 3 farm visits (interquartile range [IQR] 2–7). Of those farm visits, 255 (9.0%) involved other IPs and 2,569 (91.0%) involved 360 non-IPs (5.7% of farms in the country). The number of farm visits changed with the assumed duration of vehicle infectiousness (Figure 4), decreasing from 5 (IQR 2–9) to 2 (IQR 1–4) as the assumed duration of infectiousness decreased from 6 days to 1 day. However, the proportions of movements involving other IPs and non-IPs remained constant (Appendix Table 3). In terms of movements between IPs, 96 (37.6%) started from an IP within the 20-day period preceding the report of a suspected infection and reached another farm within 3 days, before the other farm reported a suspected infection. All these movements occurred between 5 (65.6%) IPs on Ganghwa Island or between 6 (34.4%) IPs off the island (Appendix Table 4). No vehicle movements were involved at 2 IPs (IP2 and IP11). Although another IP (IP14) was visited by such vehicles, the IP was not a source of potentially contaminating vehicle movements to other IPs, even with a vehicle infectiousness duration of 6 days (Appendix Figure 4).

### Spatial Clustering of ASF-Positive Wild Boars

**Figure 5:**
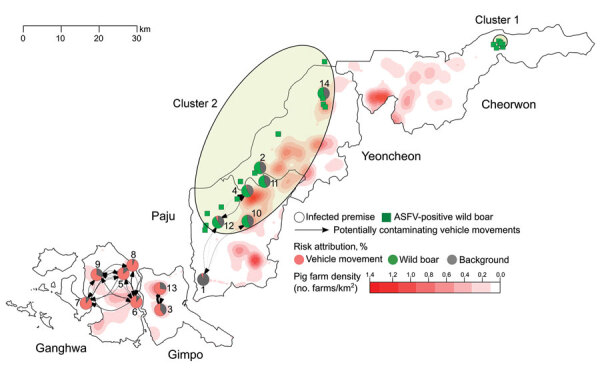
Spatial distribution of infected premises (IPs), non-IPs, African swine fever virus (ASFV)–positive wild boars, and potentially contaminating vehicle movements between IPs, South Korea, 2019. The duration of vehicle infectiousness was set to 3 days. Circles represent IPs; numbers represent the order of reporting dates. Pie charts show the estimated contribution of different transmission routes to the infection of each IP. Edge width is proportional to the number of potentially contaminating vehicle movements between IPs, weighted by the probability that an exit IP was infectious at the time of the vehicle departure. Edge arrows represent the direction of vehicle movements. Pig farm density is shown in reddish colors. Green squares represent the location of ASFV-positive wild boars; green-shaded ellipses represent spatial clusters.

During September 21–November 20 in 95 of 226 municipalities, 1,292 wild boars were tested; the rate of testing increased over time (Appendix Figure 3). A total of 26 ASFV-positive wild boars were identified in Paju (n = 6/57), Yeoncheon (n = 8/130), and Cheorwon (n = 12/398) (Figure 5). Two clusters of ASFV-positive wild boars were identified. Of 36 wild boars tested in cluster 1 (10.7 km^2^), 10 were ASFV positive, and of 131 in the larger cluster 2 (1,209.4 km^2^), 13 were positive. Wild boars caught or found dead within these clusters were 21.8 (cluster 1) and 37.2 (cluster 2) times more likely to be ASFV-positive than were those outside these clusters (p<0.001 for all). Although there was no pig farm in cluster 1, there were 6 IPs and 112 non-IPs in cluster 2 (Figure 5). The distance between an infected premise and the nearest infected wild boar was 1.3–37.0 km (Appendix Figure 5).

### The Model

For our results, we assumed that a vehicle remained infectious for 3 days after having left an IP. Changes in this parameter value did not largely affect interpretation of the results (Appendix).

When compared by using the deviance information criterion, the model accounting for vehicle movements and for exposure to wild boars in the spatial cluster was preferred over models accounting for only 1, or none, of those sources of infection (Table; Appendix Table 5). Indeed, exposure to these factors substantially increased the risk of farms becoming infected. The daily probability of infection on a farm increased 11.1-fold (95% highest density interval [HDI] 1.1–39.3) after the visit of a potentially contaminating vehicle, compared with a farm not visited by such a vehicle (Appendix Table 6). For a farm in the spatial cluster of ASFV-positive wild boars, the daily probability of becoming infected was 2.5 (95% HDI 1.0–7.7) times as high as for a farm outside this cluster (Appendix Table 6).

**Table:** Posterior parameter estimates and posterior predictive length of time between infection and reporting of African swine fever, South Korea, 2019*

Parameters	Model output		
	Median (95% HDI)	G-R	DIC
Full model			
Potentially contaminating vehicle movement (*P_v_*)	53.9 (7.4–113.4) × 10^−4^	1.00	275.8 (null model: 284.6)
Wild boar cluster (*P_w_*)	8.2 (0–19.0) × 10^−4^	1.00	
Background (country, )	0.03 (0–0.1) × 10^−4^	1.00	
Background (epidemic region, )	5.4 (1.1–11.2) × 10^−4^	1.00	
Mean of the gamma distribution (α)	3.7 (1.0–8.8)	1.00	
Variance of the gamma distribution (β)	44.6 (5.2–113.5)	1.00	
Length of time between infection and reporting (D)†	4.3 (1.0–15.8)		

*DIC, deviance information criteria; G-R, Gelman-Rubin convergence diagnostic; HDI, highest density interval; *P_v_*, risk for infection resulting from 1 potentially contaminating vehicle movement; *P_w_*, daily risk for infection resulting from being located in an African swine fever virus–positive wild boar cluster; *P_B1_*, daily background risk (country); *P_B2_*, daily background risk (epidemic region). †The distribution was obtained by simulating values from the gamma distribution, based on parameters α and β randomly sampled from their joint distribution.

**Figure 6:**
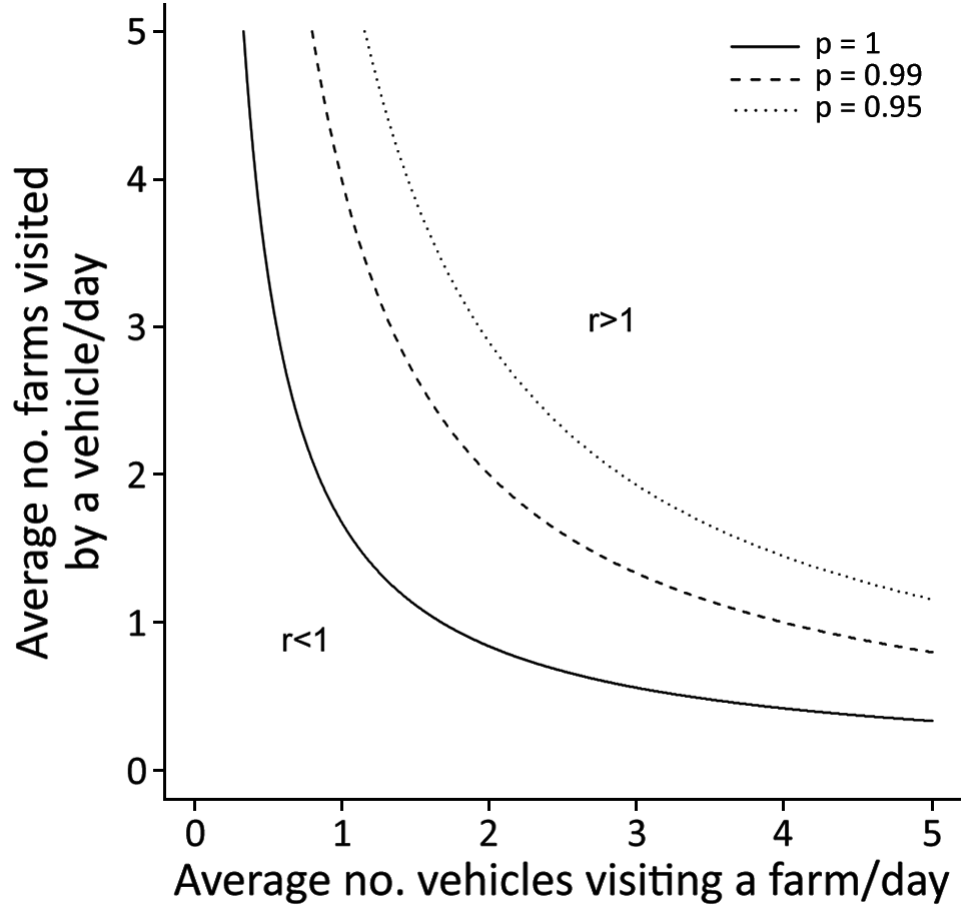
Expected number of secondary farm cases of African swine fever (r) caused by 1 infected farm through the movements of vehicles, South Korea, 2019. r is computed as a function of the average daily number of vehicles visiting a farm (x-axis) and the average daily number of farms visited by a vehicle (y-axis). Different lines represent different thresholds for the proportion of iterations in which r was <1 (p = 1, 0.99, or 0.95). Vehicles were assumed to remain infectious for 3 days after leaving an infected farm. Appendix Figure 9 shows the results with different assumptions on the duration of vehicle infectiousness.

On the basis of the best-fit model, we estimated the force of infection exerted on IPs on their estimated infection dates and the proportion of ASFV incursions attributable to each transmission route. Vehicle movements accounted for 41.2% and exposure to wild boars in the spatial cluster for 24.0% of viral incursions; the background risk accounted for the remaining 34.8% (Appendix Table 7). The contribution of different transmission routes to ASFV incursion into IPs varied with the spatial location of the farms. Vehicle movements were the most likely route for ASFV introduction into IPs in the southwestern epidemic region. In contrast, ASFV was not likely to have been spread by vehicles in the northeastern epidemic region, where wild boars were estimated to be the main source of infection for IPs within the ASFV-positive wild boar cluster (Figure 5; Appendix Figures 6,7). Indeed, the density of potentially contaminating vehicle movements differed greatly between these regions. After accounting for the posterior predictive probability that an infected premise was already infected when a vehicle left it, the estimated number of potentially contaminating vehicle movements was 36.6 between IPs and 891.6 from IPs to non-IPs (Appendix Table 8). Of those movements between IPs, 94.3% reached IPs in the southwestern (4.3 visits/infected premise) regions and 5.7% reached IPs in the northeastern (0.3 visits/infected premise) regions. Also, among farms visited by potentially contaminating vehicles, the force of infection resulting from these vehicle movements was much higher for IPs than for non-IPs (Appendix Figure 8). Together, these findings indicate that a dense network of potentially contaminating vehicle movements was formed between a small group of farms, despite the short length of time between farm infection and reporting (median 4.3 days, 95% HDI 1.0–15.8) (Table). To avoid an infected farm spreading ASFV to >1 other farm through vehicle movements, the average number of vehicles visiting a farm in a day and the average number of farms visited by a vehicle in a day should be limited to 1.3 (Figure 6).

## Discussion

Our investigation of the role of vehicle movements and of wild boars in ASFV spread to pig farms during the 2019 epidemic in South Korea was made possible by the availability of vehicle movement data generated by integrated GPS tracking and case data on wild boars generated by enhanced ASFV surveillance. The model that accounted for the influence of vehicle movements and wild boars best explained the epidemic pattern, suggesting that both transmission routes contributed to ASFV spread.

Our model suggests that the main route of ASFV introduction into IPs in the southwestern epidemic region was through contaminated vehicles. Indeed, most IPs on Ganghwa Island and Gimpo were densely connected through vehicle movements. In particular, there were a large number of vehicle movements between the 5 IPs on Ganghwa Island (IPs 5–9) ≈1–9 days before a suspected ASFV infection was reported. These 5 IPs reported possible ASF outbreaks within a 4-day period; a small number of pigs showed clinical signs at the time of reporting. This finding suggests that the high density of vehicle movements probably promoted virus transmission between these IPs. Vehicle movements may have increased ASFV spread more because of potentially less effective vehicle disinfection measures on the island. According to epidemiologic investigations, IPs on Ganghwa Island seemed to have insufficient disinfection facilities for vehicles and personnel. Moreover, although farms were relatively close together on this small island (total 302.4 km^2^), most vehicle disinfection stations were near 2 bridges connecting the island to the mainland. Therefore, vehicle movements on the island were likely to bypass these stations.

It is unclear how the virus reached the southwestern epidemic region. No potentially contaminating vehicle movements from other affected municipalities were recorded, even when the infectious period for a contaminated vehicle was extended to 6 days. No wild boars were caught or found dead, and they were therefore unavailable for ASFV testing in either municipality. Although the lack of boars for testing does not exclude the possibility that ASFV circulated in the wild boar population, the number of wild boars may be relatively small and the risk for ASFV spread from wild boars to domestic pigs may be very low in this region. Alternatively, ASFV could have been introduced through vehicle movements not captured in this study. We accounted only for vehicle movements between farms; we did not account for vehicle movements involving other types of premises (e.g., slaughterhouses) that could have acted as a source of infection.

Our results suggest that exposure to ASFV-positive wild boars was the main source of infection for pig farms in the northeastern epidemic region. First, all IPs except 1 (IP1) in Paju and Yeoncheon were located in a cluster encompassing almost all ASFV-positive wild boars found in those municipalities. Second, unlike IPs in the southwestern region, several IPs in Paju and Yeoncheon were not connected, or were only weakly connected, to other IPs through vehicle movements. However, the way in which ASFV could have spread from wild boars to domestic pigs remains unclear. Pietschmann et al. (*18*) showed that ASFV transmission was possible from wild boars to domestic pigs housed in separate pens. Such contact was, however, unlikely to have occurred in this setting because the pigs were kept indoors in all but IP11, a backyard farm. Also, potential biological vectors (*Ornithodoros* spp. ticks) have not been reported in South Korea (*19*,*20*). Although the exact mode of ASFV transmission remains unknown, the potential for ASFV spread from infected wild boars must be addressed by ASF prevention and control efforts, a view that is supported by a previous study that linked epidemics in wild boars and domestic pigs in the Russian Federation (*3*).

The nationwide GPS vehicle tracking system provided a unique opportunity to investigate the role of vehicle movements in virus dissemination between farms. Although the estimated length of time from farm infection to reporting was short and several movement restriction (standstill) periods were enforced, a large number of vehicles had already visited IPs during their estimated infectious period and could have spread ASFV to other farms. The types of vehicles and the purpose of the farm visits were not made available for this study. Vehicles involved in farming activities were required by law to be disinfected at multiple sites (e.g., the entry point of a city, town, or village) during the epidemic and routinely disinfected at the farm entrance. These findings suggest that disinfection may have been suboptimal. Therefore, restrictions on vehicle movements should be prioritized in the event of virus introduction into areas where high on-farm biosecurity cannot be guaranteed. The availability of contact tracing data could reduce the negative effect of movement restrictions on farming activities by targeting those restrictions to premises that have been in contact with IPs. Active surveillance could also be focused on these premises, enabling even more timely case detection.

The background risk accounted for a substantial fraction of the force of infection exerted on several IPs. Swill feeding probably did not contribute to this background risk because it was banned at the start of the epidemic and, according to the outbreak investigations, did not seem to be practiced on IPs. Control measures were unlikely to have promoted ASFV dissemination. Pigs were culled within a few days after confirmation of diagnostic results, and carcasses were placed inside a fiber-reinforced plastic chamber and buried on the premise. Vehicles and personnel involved in these interventions were not allowed to visit non-IPs throughout the epidemic. Regular inspections of vehicles visiting feed and manure disposal plants suggested that most vehicles involved in farming activities were registered and therefore tracked by GPS. Nonetheless, some vehicle movements not captured in this study could have contributed to disease spread. For instance, we did not consider vehicle contamination from visiting other types of premises (e.g., slaughterhouses). Private vehicles were not GPS tracked, but outbreak investigations did not identify any connections between IPs through such vehicles. Wild boars may have also substantially contributed to the background risk. Although these findings strongly suggest that the prevalence of ASFV infection in wild boars was much higher within than outside the clusters, it was not possible to exclude the possibility that the virus might also have circulated at lower prevalence in wild boar populations outside the spatial clusters. This source of infection might have been plausible for some IPs for which most of the force of infection was attributed to background risk.

One limitation of this study is that the model did not consider the possible heterogeneity in the infectiousness of vehicles and the susceptibility of farms to ASFV infection. Farm visits may involve different types of contact with persons, equipment, and pigs, thereby presenting different transmission risks. In addition, farms with poor biosecurity could have been exposed to an increased risk for infection when visited by contaminated vehicles. The risk for infection from infected wild boars was also likely to have varied between farms because of different levels of on-farm biosecurity and proximity to wild boar habitats. In addition, although the model identified an excess risk for infection for farms within the spatial cluster of ASFV wild boar cases, the spatiotemporal heterogeneity in ASFV circulation among wild boars inside and outside the cluster may have been underestimated.

Another limitation is that the model assumed that the potential for a contaminated vehicle to transmit the infection remained constant throughout the vehicle’s period of infectiousness. Yet because vehicles were supposed to be disinfected when entering a farm, their infectiousness may have decreased over time with each additional farm visited. Accounting for this process would probably have increased the estimated probability of virus incursion after a visit from a potentially contaminating vehicle. However, this process is unlikely to have influenced our conclusions because the contribution of vehicle movements to ASFV spread was not affected by variations in the assumed duration of vehicle infectiousness.

The possibility that some wild boars were infected while on IPs cannot be completely excluded. However, ASFV was probably circulating among wild boars before pig farms were infected, given that the first wild boar case was detected in the demilitarized zone where no civilians and farms are present, and North Korea had already reported the disease. Subsequently, the delayed detection of ASFV in wild boars probably resulted from the lower sensitivity of surveillance in wild animals compared with domestic animals and from increased surveillance efforts among wild boars after disease detection on farms. In addition, since the end of the study period (November 21, 2019), ASFV infection has been confirmed in >700 wild boars and on 3 pig farms (*2*), suggesting that ASFV can continue to circulate among wild boars in the absence of virus circulation among domestic pigs.

Our models did not account for within-farm transmission dynamics. Farm infectiousness was likely to vary over time, influencing between-farm transmission dynamics. However, given that a small number of pigs showed ASF-like clinical signs on all IPs at the time of reporting or detection, and that herds were culled within 1 or 2 days, the effect may have been limited.

In conclusion, our findings suggest that the movement of contaminated vehicles and infected wild boars contributed to the spread of ASFV to pig farms in South Korea. Although the ongoing circulation of ASFV in wild boars poses an ongoing risk for virus spillover onto pig farms, vehicle movements have the potential to cause large chains of transmission between farms. Therefore, the timely implementation of movement restrictions is critical for the rapid and effective management of ASFV epidemics. In this regard, the tracking of vehicles involved in farming activities could guide the targeting of restrictions to those at high risk for infection because of their recent contacts. High on-farm biosecurity and effective vehicle disinfection should be ensured, especially in areas where ASFV is circulating among wild boars.

Appendix Additional methods and results for study of transmission dynamics of African swine fever virus, South Korea, 2019.
