# Supplementary material for: Transmission Dynamics of African Swine Fever Virus, South Korea, 2019
Source: Emerg Infect Dis. 2021 Jul;27(7):1909–18. doi: 10.3201/eid2707.204230 (PMC8237864; doi:10.3201/eid2707.204230)
Supplement: Appendix — Additional methods and results for study of transmission dynamics of African swine fever virus, South Korea, 2019. [file 20-4230-Techapp-s1.pdf]

Article DOI: https://doi.org/10.3201/eid2707.204230

Transmission Dynamics of African Swine Fever Virus, South Korea, 2019

Appendix

Methods

1. Between-farm vehicle movements

Vehicle movement data were collated from the Korean Animal Health Information System (KAHIS). By law, vehicles used for livestock farming activities, such as veterinary medicine, feed, manure, livestock transport, and used by governmental veterinary services (e.g. for disease surveillance and control), were required to be installed with a GPS device. It was not required for private vehicles due to the personal information protection law. It transmitted a signal to KAHIS when a vehicle was located on the site of a farm registered in the government database. As of 2019, 59,521 vehicles were registered in KAHIS, and, based on results of regular inspections, it could be assumed that the vast majority of vehicles were registered (Yoo, personal communication). In particular, close to 100% of veterinary services vehicles and feed and manure transporters, for which their registration was inspected each time they enter feed or manure disposal plants, were considered to be enrolled.

Vehicle movements between farms were identified in two steps. First, we extracted from KAHIS all vehicle movements made by vehicles that entered IPs during the study period: from 28th August 2019 (20 days before the first IP report) to 16th October 2019 (one week after the last IP report). The definition of the study period was based on the following assumptions. Considering that the number of pigs showing clinical signs indicative of ASF was small, zero to five, at the time of reporting, and that the ASF incubation period in domestic pigs is estimated to be around four to 13 days (*1*), we assumed that the length of time between infection and reporting was less than 20 days during this epidemic. We also assumed that farms were infectious from the onset of infection, and remained infectious after reporting, until the end of the epidemic, recognizing that vehicles that entered IPs for sample collection, culling and

disinfection could have become contaminated with ASFV from infected pigs and the contaminated environment. Second, from the extracted data, we identified between-farm vehicle movements that could have played a role in the spread of ASFV. We assumed that a vehicle contaminated following the visit of an infected farm was infectious for other farms for a certain time period. Thus, we assumed that a vehicle *v* could have spread the virus from farm *i* to farm *j* if it entered farm *j* within *d* days after having visited farm *i* during the infectious period of that farm. We defined vehicle movements satisfying this condition as 'potentially contaminating vehicle movements, V'. Such movements were not limited to successive visits. We assumed that vehicle *v* could infect farm *j*, regardless of whether it entered other farms during these *d* days. For example, if contaminated vehicle *v* entered susceptible farms *j* and then *k* within *d* days after leaving infectious farm *i*, the vehicles could have infected farm *j* as well as farm *k*, with the probability of infecting farm *k* being independent of whether farm *j* became infected. ASFV is known to be persistent, with the ability to survive for several weeks in the environment (*2*). However, considering that vehicles are required by law to be disinfected before entering farms, and were disinfected when entering or exiting a city, town, or village during the epidemic, we assumed that contaminated vehicles could not remain infectious for longer than a week. In the following, we considered different values of *d*, the number of days during which a contaminated vehicle remained infectious: one, three and six days.

2. Surveillance data on ASF in wild boars and on pig farms

The Ministry of Environment reinforced ASF surveillance in wild boars by providing financial incentives for wild boar hunting, trapping and carcass reporting. All wild boars caught or found dead across the country were tested for ASFV. The Ministry of Environment provided ASFV test results (i.e. positives or negatives) and the spatial coordinates where the tested wild boars were caught or found dead. We assessed whether wild boar cases were spatially clustered using an elliptic version of the spatial scan statistic in SatScan software (https://www.satscan.org). The cluster with the largest maximum likelihood ratio test statistic represented the most likely cluster of ASFV-positive wild boars, and secondary clusters of ASFV-positive wild boars were identified based on the iterative scan statistics (https://www.satscan.org). The statistical significance of detected clusters was assessed by comparing the test statistic of the observed data with those of data randomly generated by 999 Monte Carlo simulations. The p-values were calculated using the default SaTScan option

(https://www.satscan.org). We set the maximum cluster size to 50% of the population at risk for the following reasons. First, clusters obtained with >50% sizes indicate areas of exceptionally low rates of wild boar cases within the defined cluster, rather than an area of exceptionally high rates of wild boar cases within the cluster (https://www.satscan.org). Thus, 50% is generally recommended as an upper limit (https://www.satscan.org), to consider all possible sizes of clusters below this value. Secondly, restricting cluster sizes below 50% could introduce pre-selection bias in the cluster size unless there was solid epidemiological reason to choose such lower values (https://www.satscan.org).

3. Bayesian modelling approach

We hypothesized that vehicle movements from IPs and ASFV-infected wild boars were the main sources of infection for domestic pig farms. To test this hypothesis, we formulated the force of infection based on four parameters: P_V was the risk of infection resulting from one potentially contaminating vehicle movement, P_W was the daily risk of infection resulting from being located in an ASF-positive wild boar cluster. P_{B_1} and P_{B_2} were the daily risks of infection not captured by P_V and P_W. While P_{B_1}represented a background risk for all farms in the country, P_{B_2} represented an additional background risk for farms in the epidemic region.

The force of infection for farm *i* on day d ($F_{i,d}$) was then modelled as:

$$F_{i,d} = P_V n_{i,d}^V + P_W S_i^W + P_{B_1} + P_{B_2} A_i$$

where $n_{i,d}^V$ was the number of potentially contaminating vehicle movements farm *i* received on day *d* and S_i^W and A_i were an indicator variable ($S_i^W = 1$ if farm *i* was located in the spatial cluster, 0 otherwise, $A_i = 1$ if farm *i* was located in the epidemic region, 0 otherwise).

Since infection dates were not observed for IPs, we updated infection dates (I_i), and therefore the time between infection and reporting (D_i), in each iteration by using a data augmentation technique, which has been successfully adopted to infer transmission dynamics from incomplete epidemic data (*4*). The likelihood of the epidemic data was expressed as follows. As starting values, we randomly selected the time between infection and reporting from a Uniform distribution between 1 and 20 days for each IP, and computed their infection date. For an IP *I*, the time between infection and reporting (D_i) and its augmented infection date (I_i) were:

$D_i \sim \mathrm{U}(1, 20)$, $D_{set} = \{D_1, \dots, D_{14}\}$

$I_i = R_i - D_i, I_{set} = \{I_1, \dots, I_{14}\}$

R_i was the date at which IP *i* reported suspicion of ASFV infection. With I_{set} as starting values, the probability A_i that IP *i* was not infected between the start of the epidemic and one day before its augmented infection date, I_i, and the probability B_i that IP *i* was infected on day I_i were expressed as follows:

$A_i = e^{\sum_{d=1}^{I_i - 1} -F_{i,d}}, B_i = 1 - e^{-F_{i,I_i}}$

Note that $n_{i,d}^V$ and, therefore, $F_{i,d}$, were updated based on I_{set}. The likelihood L_1 for the timing of infection of IPs was:

$$L_1 = \prod_{i, i \in F_{IP}} A_i B_i f(D_i; \alpha, \beta)$$

F_{IP} refers to the set of IPs. $f(D_i; \alpha, \beta)$ was the probability density function of the length of time between infection and reporting in farms. It was expressed as a Gamma distribution, with α and β as mean and variance. The Gamma distribution was truncated to between 1 and 20, as it was assumed that the time between infection and reporting. For non-IPs, we expressed the probability that farm *j* did not become infected during the epidemic (C_j) and the likelihood for non-IPs (L_2) as:

$$C_j = e^{\sum_{d=1}^{d_{last_j}} -F_{j,d}}$$

$$L_2 = \prod_{j, j \notin F_{IP}} C_j$$

Farms which were depopulated by culling or government purchase were no longer at risk of infection. Thus, for those farms, d_{last_i} was defined as the date at which farm *i* was emptied during the epidemic. For farms that were not subject to those preventive measures, d_{last_i} was the last date of the study period.

The likelihood of the epidemic data *L* was:

$$L = L_1 L_2$$

At the first stage of the MH algorithm, new values were proposed for the parameters related to transmission (P_V, P_W and P_B) and the time between infection and reporting (α and β). For each parameter *i*, a new value was proposed from the Uniform distribution:

$$\theta_i^{new} \sim U(\theta_i^{old} - \varepsilon_i, \theta_i^{old} + \varepsilon_i)$$

θ_i^{new} was the proposed value in the current iteration, θ_i^{old} was the value in the previous iteration, and $\boldsymbol{\varepsilon_i}$ was a scale parameter. We constrained P_V, P_W, P_{B_1}, and P_{B_2} to be between 0 and 1. Therefore, when values outside this range were proposed, they were discarded, and new values were proposed. Then, for the transmission and Gamma distribution parameters, the proposed values were accepted based on the following acceptance ratio:

$$\frac{Posterior(\theta^{new}|y, D, I)}{Posterior(\theta^{old}\ |y, D, I)} \wedge 1$$

θ^{new} was as a set of proposed values in the current iteration, θ^{old} was as a set of parameter values in the previous iteration, and $\boldsymbol{y}$ was the data. If the acceptance ratio was equal to or greater than a random number drawn between 0 and 1, θ^{new} was accepted. Otherwise, θ^{new} was discarded, and θ^{old} was recycled in the next iteration. While the transmission parameters were proposed and accepted (or discarded) separately, the Gamma distribution parameters were proposed and accepted (or discarded) together. Additionally, for effective MCMC mixing, for a set of proposed values *i*, the scale parameter $\boldsymbol{\varepsilon_i}$ was increased or decreased by 20% in every 100th iteration if the acceptance rate fell below 20% or exceeded 30% (Appendix Table 1).

For a given iteration, once parameter values were updated, we also updated the time between infection and reporting (and therefore infection dates) for each IP, one by one, by using an independence MH sampler. For IP *i*, we proposed an integer value randomly drawn from a Uniform distribution between 1 and 20 days as IP *i*'s new value for the time between infection and reporting (D_i^{new}).

$$D_i^{new} \sim \mathrm{U}(1, 20)$$

$$D_{set}^{new} = D_{set}^{old} - \{D_i^{old}\} + \{D_i^{new}\}$$

We also proposed a new infection date for IP *i* ($\boldsymbol{I_i^{new}}$) as:

$$I_i^{new} = R_i - D_i^{new}$$

$$I_{set}^{new} = I_{set}^{old} - \{I_i^{old}\} + \{I_i^{new}\}$$

Then, we updated $n_{i,d}^V$ with I_{set}^{new} and decided whether to accept D_{set}^{new} and I_{set}^{new} based on the following acceptance ratio:

$$\frac{Posterior(D_{set}^{new}, I_{set}^{new}|y,\theta)}{Posterior(D_{set}^{old}, I_{set}^{old}\ |y,\theta)} \hat{}1$$

If the acceptance ratio was equal or greater than a random number drawn between 0 and 1, D_{set}^{new} and I_{set}^{new} were accepted. Otherwise, D_{set}^{old} and I_{set}^{old} were recycled in the next iteration. Weakly informative priors were used for model parameters (Appendix Table 2).

The models were iterated up to the point where convergence was considered to have been achieved based on a visual inspection of MCMC trace plots and Gelman-Rubin convergence diagnostic. The models were run with four chains with different starting values. The first 10,000 iterations were discarded, and the remaining parameter values comprised the posterior distributions. We estimated the posterior predictive distribution of the length of time between infection and reporting through simulations based on the joint posterior distribution of α and β. We compared models based on their DIC to assess whether the model accounting for both the influence of vehicle movements and wild boars explained the epidemic pattern better than models accounting for the influence of only one of these epidemiological factors, or only the constant background risk (null model). Since infection dates were not observed, the conventional DIC could not be used. A modified version of DIC designed for models with missing data was used instead (*3*). Since the posterior distribution was right-skewed for some parameters, the posterior median, instead of the posterior mean, was reported and used to compute DIC values (*4*).

Based on the joint posterior distribution of the parameters, we computed the expected number of secondary farm cases generated by one infected farm through the movement of vehicles, for different values of the average daily numbers of (i) farms visited by a vehicle ($n_{v\rightarrow f}$) and (ii) vehicles visiting a farm ($n_{f\leftarrow v}$):

$$n_{v\rightarrow f} n_{f\leftarrow v} C_v I_f P_v$$

C_v was the assumed duration of vehicle infectiousness, I_f was the average infectious period of a farm, and P_v was the risk of infection resulting from one potentially contaminating vehicle movement.

Next, we computed RR_V (or RR_W), the ratio between the daily probability of a farm becoming infected if it received one potentially contaminating vehicle movement (or if it was located in the spatial cluster of ASFV-positive wild boars) and the daily probability of a farm becoming infected from transmission routes other than vehicle movements and wild boars. With $P_B = P_{B1} + P_{B2}$:

$$RR_V = (1 - e^{-(P_V + P_B)})/(1 - e^{-P_B})$$

$$RR_W = (1 - e^{-(P_W + P_B)})/(1 - e^{-P_B})$$

We also estimated for individual IPs the force of infection on their estimated dates of infection, and the proportion of ASFV infection attributable to different transmission routes, through simulation. In each iteration, we randomly sampled parameter values $(P_V, P_W, P_B, \alpha, \text{and } \beta)$ from the joint posterior distributions. Then, based on the Gamma distribution with the sampled parameters α and β as mean and variance, we computed the probability that IP *i* was infected on day *d*, $\delta(i, d)$, and the probability that IP *j* was infectious on day *s*, $\pi(j, s)$. If vehicle *v* left IP *j* on day *s* and visited IP *i* on day *d*, we computed the force of infection resulting from that vehicle movement ($F_{v,j,s,i,d}$) as follows:

$$F_{v,j,s,i,d} = \delta(i, d)\pi(j, s)P_V I(s, d)$$

Where $I(s, d)$ was equal to 1 if the difference between days *s* and *d* was lower than or equal to the assumed length of the vehicle infectious period, and 0 if not. $F_{V,i}$ was then the force of infection resulting from vehicle movements exerted on IP *i* when this IP was estimated to have been infected:

$$F_{V,i} = \sum_{v,j,s,d} F_{v,j,s,i,d}$$

The force of infection associated with wild boars was $F_{W,i} = P_W$ if IP *i* was located in the cluster, and $F_{W,i} = 0$ otherwise. The force of infection associated with the background infection risk was $F_{B,i} = P_B$. In each iteration (i.e. for each set of parameters sampled from the joint

posterior distribution), we also randomly assigned a source of infection to each IP i by simulating a multinomial trial, with one trial and 3 possible outcomes (i.e. each transmission route) associated with probabilities $F_{V,i}/K$, $F_{W,i}/K$, and $F_{B,i}/K$, with $K = F_{V,i} + F_{W,i} + F_{B,i}$. We repeated this process 30,000 times, and computed the proportion of simulated infections attributed to each route of transmission.

We estimated the weighted number of potentially contaminating vehicle movements between IPs. In each iteration (i.e. a set of parameter values drawn from the joint posterior distribution), we identified all vehicle movements made within the duration of the assumed vehicle infectious period and before entry IPs (i.e., IPs where vehicles moved to) reported a suspicion of ASFV infection. Then, we weighted these vehicle movements by the probability that exit IPs (i.e., IPs that vehicles had visited) were infectious on the day of departure, based on randomly sampled Gamma distribution parameter values. We repeated this process 30,000 times.

Finally, we estimated the force of infection resulting from potentially contaminating vehicle movements for farms associated with such vehicle movements. If vehicle *v* had visited IP *i* and day *s* and farm *i* on day *d*, we computed the force of infection exerted on farm *j* resulting from that vehicle movement ($F_{v,j,s,d}$) as follows:

$$F_{v,j,s,i,d} = \pi(j,s)P_V I(s,d)$$

We then computed the overall force of infection exerted on farm *i* resulting from potentially contaminating vehicle movements (F_j) as follows:

$$F_i = \sum_{v,j,s,d} F_{v,j,s,i,d}$$

We compared F_j between farms within and outside affected municipalities.

References

1. Guinat C, Reis AL, Netherton CL, Goatley L, Pfeiffer DU, Dixon L. Dynamics of African swine fever virus shedding and excretion in domestic pigs infected by intramuscular inoculation and contact transmission. Vet Res (Faisalabad). 2014;45:93. PubMed https://doi.org/10.1186/s13567-014-0093-8

2. Davies K, Goatley LC, Guinat C, Netherton CL, Gubbins S, Dixon LK, et al. Survival of African swine fever virus in excretions from pigs experimentally infected with the Georgia 2007/1 isolate. Transbound Emerg Dis. 2017;64:425–31. PubMed https://doi.org/10.1111/tbed.12381

3. Celeux G, Forbes F, Robert CP, Titterington DM. Deviance information criteria for missing data models. Bayesian Anal. 2006;4:651–74.

4. Walker PG, Cauchemez S, Métras R, Dung H, Pfeiffer D, Ghani AC. A Bayesian approach to quantifying the effects of mass poultry vaccination upon the spatial and temporal dynamics of H5N1 in northern Vietnam. PLOS Comput Biol. 2010;6:e1000683. PubMed https://doi.org/10.1371/journal.pcbi.1000683

Appendix Table 1. Initial scale parameter values

Parameters		Initial scale parameter values
Force of infection parameters	P_V	0.01
	P_W	0.001
	P_{B_1}	0.00001
	P_{B_2}	0.0001
Gamma distribution parameters*	α	3
	β	3

* For effective MCMC mixing, the scale parameters were increased or decreased by 20% in every 100th iteration if the acceptance rate fell below 20% or exceeded 30%

Appendix Table 2. Priors for the transmission and Gamma distribution parameters

Parameters		Priors
Transmission parameters	P_V	Uniform (0, 1)
	P_W	Uniform (0, 1)
	P_{B_1}	Uniform (0, 1)
	P_{B_2}	Uniform (0, 1)
Gamma distribution parameters*	α	Uniform (1, 20)
	β	Gamma (mean = 6.5, variance = 2)

*α and β were the mean and variance of the Gamma distribution of the time between infection and reporting.

Appendix Table 3. The pattern of vehicle movements between pig farms

Duration of vehicle infectiousness*	No. of vehicles	No. of vehicle movements	Between IPs	IPs to non-IPs	No. of non-IPs†
One day	122	1148	107 (9.3%)	1041 (90.7%)	182 (2.9%)
Three days	156	2824	255 (9.0%)	2569 (91.0%)	360 (5.7%)
Six days	169	4115	355 (8.6%)	3760 (91.4%)	479 (7.6%)

*Vehicle movements were defined as those made between two pig farms within the assumed duration of vehicle infectiousness.
†The number of non-IPs that received at least one vehicle movement among the study population (n=6,340)

Appendix Table 4. The pattern of potentially contaminated vehicle movements between IPs*

	No. of potentially contaminated vehicle movements		
Movement direction	One day†	Three days†	Six days†
Inside Ganghwa	37 (5)	63 (5)	85 (5)
Outside Ganghwa	10 (5)	33 (6)	46 (7)
From Ganghwa to other municipalities	0	0	0
From other municipalities to Ganghwa	0	0	0
Total	47	96	131

*Vehicle movements made between IPs up to 20 days before exit IPs (i.e. IPs where vehicles moved from) reported suspicion of infection and before entry IPs (i.e. IPs where vehicles moved to) reported suspicion of infection, with different assumptions on the duration of vehicle infectiousness (i.e. one, three, or six days)
†The assumed duration of vehicle infectiousness
The number in brackets represents the number of IPs involved with given vehicle movements.

Appendix Table 5. Posterior parameter estimates with the three-day assumption on the duration of vehicle infectiousness

	MCMC output		
Parameters	Median (95% HDI*)	G-R†	DIC‡
Full model			
Potentially contaminating vehicle movement (P_V)	53.9 (7.4-113.4) x10^{-4}	1.00	275.8
Wild boar cluster (P_W)	8.2 (0-19.0) x10^{-4}	1.00	
Background (country, P_{B_1})	0.03 (0-0.1) x10^{-4}	1.00	
Background (epidemic region, P_{B_2})	5.4 (1.1-11.2) x10^{-4}	1.00	
Mean of the Gamma distribution (α)	3.7 (1.0-8.8)	1.00	
Variance of the Gamma distribution (β)	44.6 (5.2-113.5)	1.00	
‘Vehicle movement’ model			
Potentially contaminating vehicle movement (P_V)	50.1 (5.9-110.8) x10^{-4}	1.00	277.8
Background (country, P_{B_1})	0.03 (0-0.1) x10^{-4}	1.00	
Background (epidemic region, P_{B_2})	8.3 (3.5-14.2) x10^{-4}	1.00	
Mean of the Gamma distribution (α)	3.8 (1.0-9.1)	1.00	
Variance of the Gamma distribution (β)	45.2 (5.5-114.1)	1.00	
‘ASFV-circulation in wild boars’ model			
Wild boar cluster (P_W)	5.6 (0-16.1) x10^{-4}	1.00	285.8
Background (country, P_{B_1})	0.03 (0-0.1) x10^{-4}	1.00	
Background (epidemic region, P_{B_2})	9.8 (4.4-16.2) x10^{-4}	1.00	
Mean of the Gamma distribution (α)	4.7 (1.0-17.9)	1.00	
Variance of the Gamma distribution (β)	44.4 (6.1-113.6)	1.00	
Null Model			
Background (country, P_{B_1})	0.03 (0-0.1) x10^{-4}	1.00	284.6
Background (epidemic region, P_{B_2})	11.5 (6.2-17.7) x10^{-4}	1.00	
Mean of the Gamma distribution (α)	4.6 (1.0-17.7)	1.00	
Variance of the Gamma distribution (β)	4.5 (6.4-115.0)	1.00	

*Highest density interval
†Gelman-Rubin convergence diagnostic
‡The deviance information criteria. DIC in Celeux, et al. (*3*) was used.

Appendix Table 6. The probability ratios, based on posterior parameter estimates from the full model

Probability ratios	Median (95% HDI*)
Duration of vehicle infectiousness: one day	
Potentially contaminating vehicle movements vs no vehicle movements	23.6 (1.5-89.0)
Within vs outside the ASF-positive wild boar cluster	2.5 (1.0-7.7)
Duration of vehicle infectiousness: three days	
Potentially contaminating vehicle movements vs no vehicle movements	11.1 (1.1-39.3)
Within vs outside the ASF-positive wild boar cluster	2.5 (1.0-7.7)
Duration of vehicle infectiousness: six days	
Potentially contaminating vehicle movements vs no vehicle movements	7.5 (1.0-26.1)
Within vs outside the ASF-positive wild boar cluster	2.4 (1.0-7.4)

*Highest density interval

Appendix Table 7. The percentage of simulated infections caused by different transmission routes, based on posterior parameter estimates from the full model

Transmission route	Risk attribution (%)
Duration of vehicle infectiousness: one day	
Potentially contaminating vehicle movement	37.1
Wild boar cluster	23.8
Background (country)	0.3
Background (epidemic region)	38.9
Duration of vehicle infectiousness: three days	
Potentially contaminating vehicle movement	41.2
Wild boar cluster	24.0
Background (country)	0.2
Background (epidemic region)	34.6
Duration of vehicle infectiousness: six days	
Potentially contaminating vehicle movement	39.4
Wild boar cluster	23.2
Background (country)	0.2
Background (epidemic region)	37.2

*The number of vehicle movements was weighted by the probability that exit IPs (i.e. IPs where a vehicle moved from) were already infected on the day of departure, based on the posterior distribution of the length of time between infection and reporting.

Appendix Table 8. The weighted number of potentially contaminating vehicle movements, based on posterior parameter estimates from the full model*

Movement pattern	Median (95% quantile)
Duration of vehicle infectiousness: one day	
Between IPs	18.2 (14.4-25.0)
IPs to non-IPs	367.2 (330.9-429.9)
Duration of vehicle infectiousness: three days	
Between IPs	36.6 (28.6-52.1)
IPs to non-IPs	891.6 (794.9-1,078.0)
Duration of vehicle infectiousness: six days	
Between IPs	41.2 (31.4-66.8)
IPs to non-IPs	1226.2.0 (1104.9-1539.7)

*The number of vehicle movements was weighted by the probability that exit IPs (i.e. IPs where a vehicle moved from) were already infected on the day of departure, based on the posterior predictive distribution of the length of time between infection and reporting.

Appendix Table 9. The weighted number of potentially contaminating vehicle movements between IPs, based on posterior parameter estimates from the full model*

Movement pattern	Median (95% quantile)
Duration of vehicle infectiousness: one day	
Inside wild boar cluster	0.5 (0.2-0.8)
Outside wild boar cluster	17.7 (14.2-24.2)
Duration of vehicle infectiousness: three days	
Inside wild boar cluster	2.1 (1.1-3.6)
Outside wild boar cluster	34.5 (27.4-48.6)
Duration of vehicle infectiousness: six days	
Inside wild boar cluster	2.7 (1.4-5.4)
Outside wild boar cluster	38.5 (29.9-61.3)

*The number of vehicles leaving an IP was weighted by the probability that the IP was already infected on the day of departure, based on the posterior predictive distribution of the length of time between infection and reporting.

Appendix Table 10. Posterior parameter estimates and posterior predictive length of time between infection and reporting, from the full model with different assumptions on the duration of vehicle infectiousness

	MCMC output		
Parameters	Median (95% HDI*)	G-R†	DIC‡
Duration of vehicle infectiousness: one day			
Potentially contaminating vehicle movement (P_V)	121.8 (19.1-260.8) x10^{-4}	1.00	271.5 (null model:284.6)
Wild boar (P_W)	8.0 (0-18.9) x10^{-4}	1.00	
Background (country, P_{B_1})	0.03 (0-0.1) x10^{-4}	1.00	
Background (epidemic region, P_{B_2})	5.4 (1.0-11.3) x10^{-4}	1.00	
Mean of the Gamma distribution (α)	3.4 (1.0-8.3)	1.00	
Variance of the Gamma distribution (β)	47.1 (7.3-118.9)	1.00	
Length of time between infection and reporting (D)§	4.2 days (1.0-16.0)		
Duration of vehicle infectiousness: six days			
Potentially contaminating vehicle movement (P_V)	36.6 (3.4-79.3) x10^{-4}	1.00	280.1 (null model:284.6)
Wild boar (P_W)	8.0 (0-18.9) x10^{-4}	1.00	
Background (country, P_{B_1})	0.03 (0-0.1) x10^{-4}	1.00	
Background (epidemic region, P_{B_2})	5.7 (1.0-11.6) x10^{-4}	1.00	
Mean of the Gamma distribution (α)	3.9 (1.0-9.8)	1.00	
Variance of the Gamma distribution (β)	45.7 (6.1-115.0)	1.00	
Length of time between infection and reporting (D)§	4.5 days (1.0-16.1)		

*Highest density interval
†Gelman-Rubin convergence diagnostic
‡The deviance information criteria. DIC in Celeux, et al. (*3*) was used.
§The distribution was obtained by simulating values from the Gamma distribution, based on randomly sampled gamma distribution parameters (α and β).

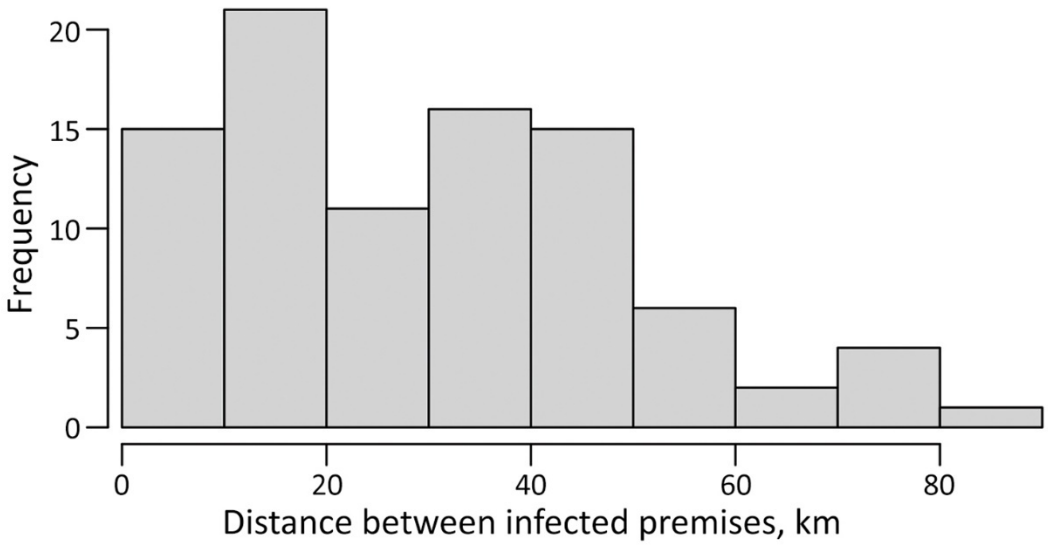

Appendix Figure 1. The distribution of the shortest distance between any two IPs. The shortest distance was computed by using the *distm* function of the *geosphere* package in R.3.4.2.

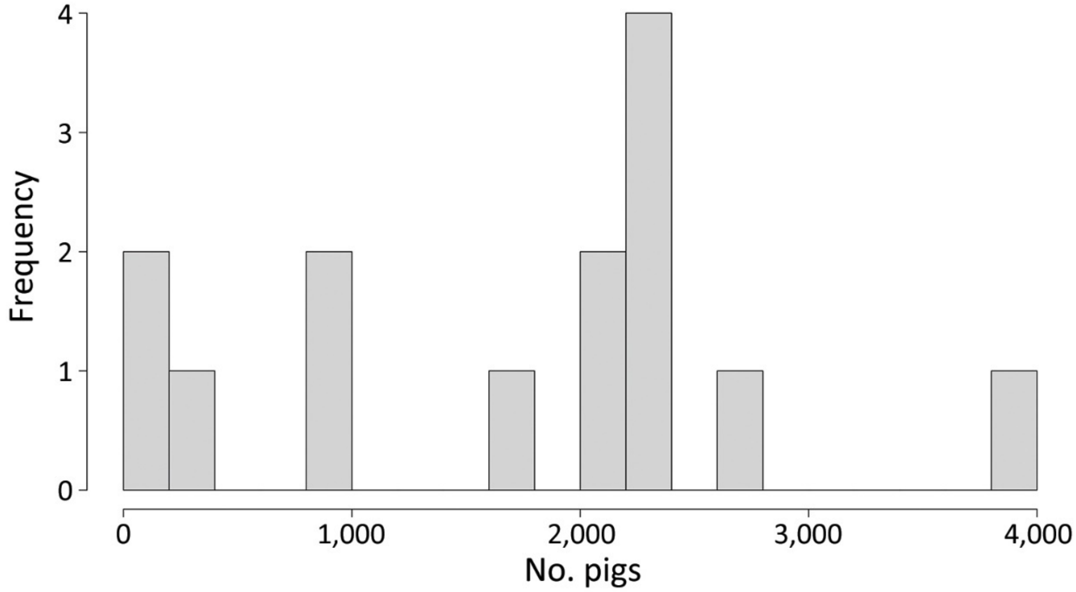

Appendix Figure 2. Histogram of the number of pigs on IPs.

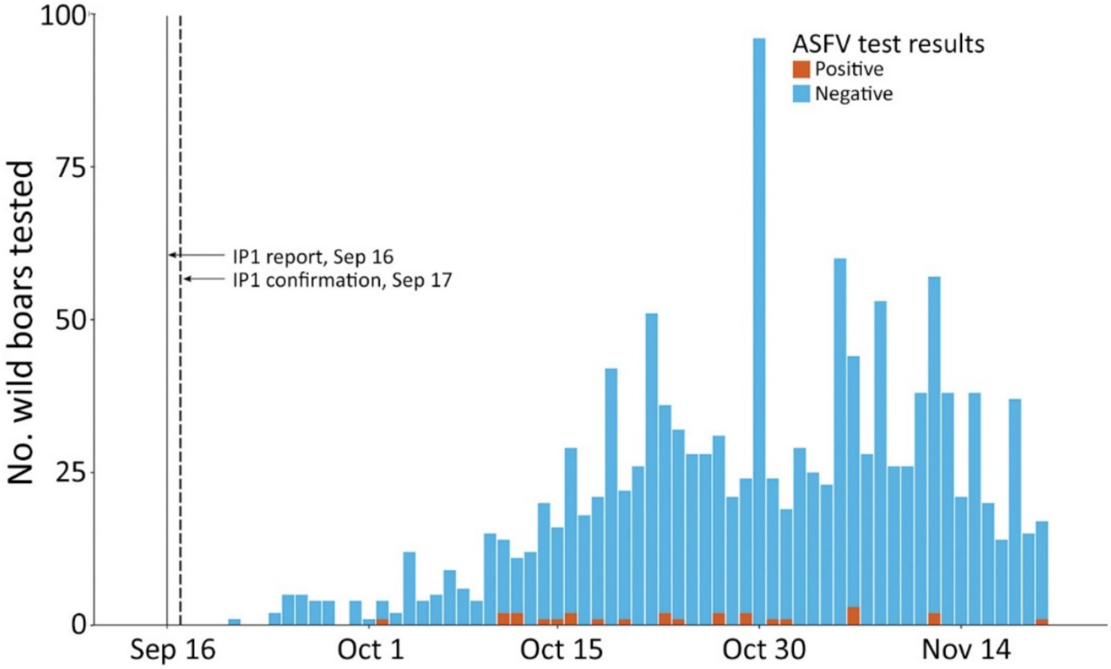

Appendix Figure 3. The number of wild boars that tested positive or negative for ASFV by RT-PCR during the study period. Since the first confirmation of ASFV infection in a domestic pig farm (IP1) on 17th September, ASF surveillance in wild boars has been intensified across the country, resulting in a total of 26 ASFV-positive wild boar out of 1,292 wild boars tested during the study period.

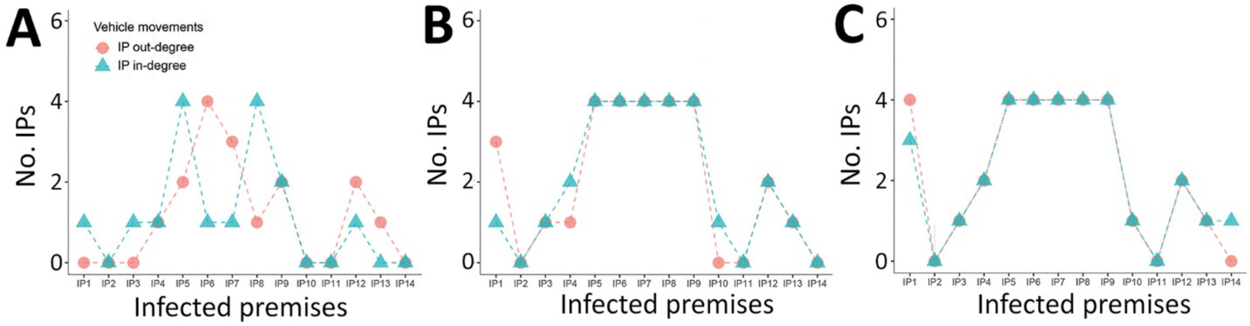

Appendix Figure 4. The distribution of the number of IPs connected through vehicle movements. With (A) one-, (B) three- or (C) six-day assumptions for the duration of vehicle infectiousness, only the movements made up to 20 days before an exit farm (i.e., farms where a vehicle moved from) reported suspicion of ASFV infection and before an entry farm (i.e., farms where a vehicle moved to) reported the suspicion are shown. For each IP (x-axis), a circle (or a triangle) represents the number of other IPs, an IP sent (or received) at least one vehicle movement (y-axis), with different assumptions for vehicle infectiousness.

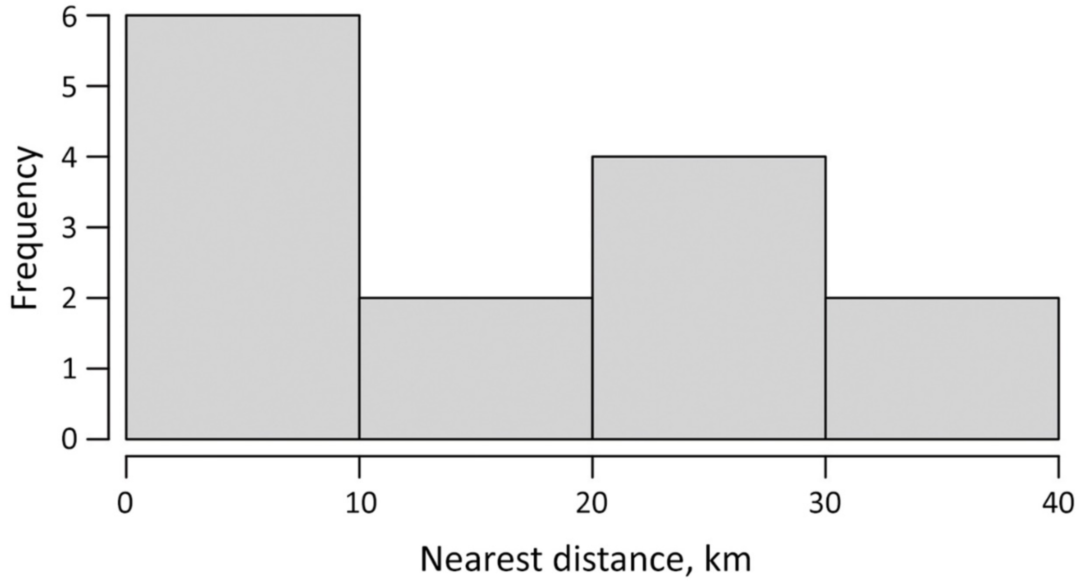

Appendix Figure 5. The distribution of the distance to the nearest location of an ASFV-positive wild boar from individual IPs. The shortest distance was computed by using the *distm* function of the *geosphere* package in R.3.4.2.

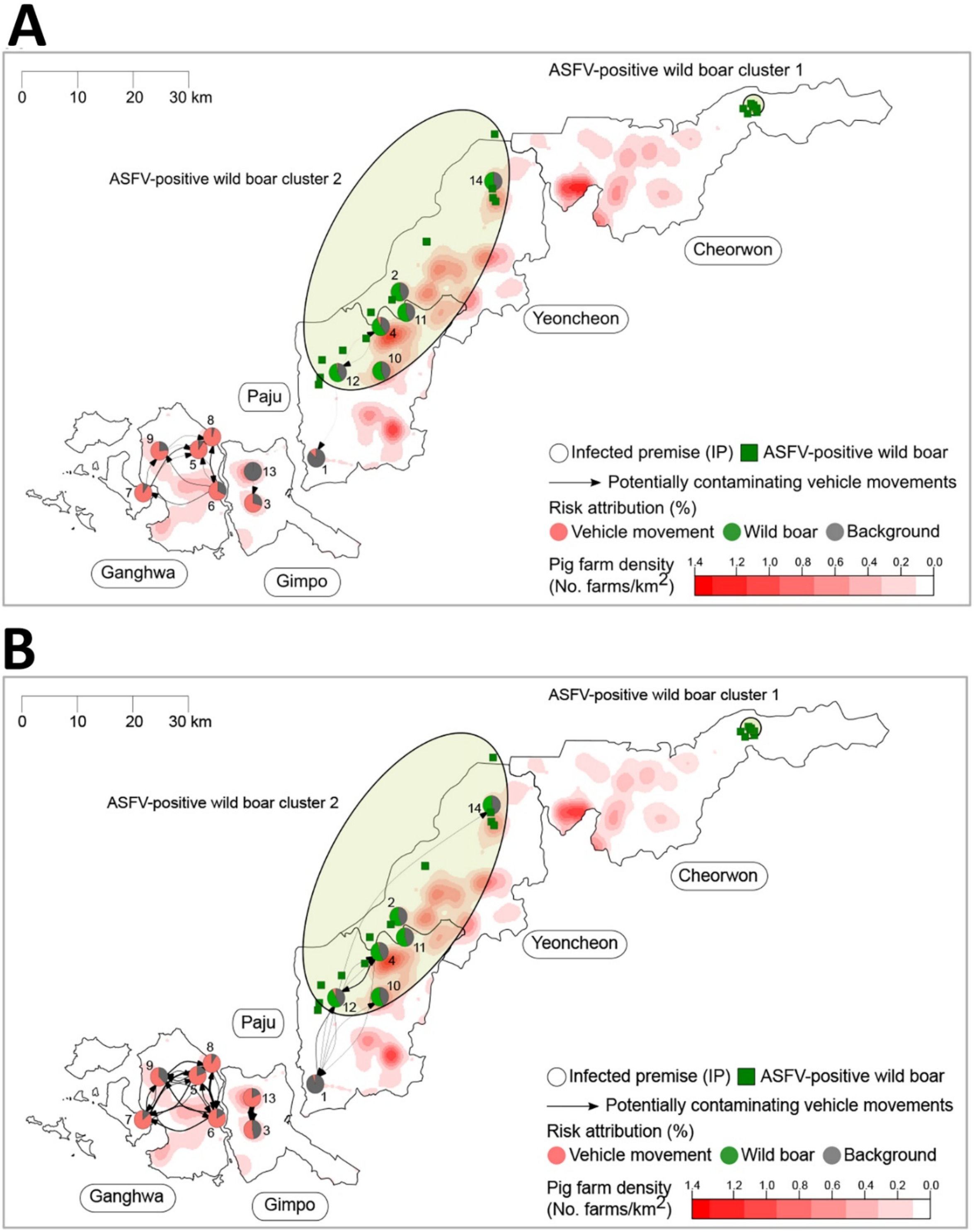

Appendix Figure 6. The vehicle movement pattern and model results. The spatial distribution of IPs, non-IPs, ASFV-positive wild boars, and potentially contaminating vehicle movements between IPs with (A) one- and (B) six-day assumptions for the duration of vehicle infectiousness. Polygons represent municipalities affected by ASFV. Small circles represent IPs, with their numbers representing the order of

reporting dates. Those circles are represented as pie charts, showing the proportion of different transmission routes attributed to the infection of an IP via simulation. Edges represent vehicle movements made between IPs when an exit IP (i.e., where a vehicle moved from) was considered infectious via simulation, and before an entry IP (i.e., where a vehicle moved to) reported suspicion of infection. Edge width is proportional to the sum of the weighted number of such movements; each vehicle movement was weighted by the probability that an exit IP was infectious at the time of departure computed via simulation. Edge arrows represent the direction of vehicle movements. Pig farm density is shown in reddish colors. Green squares represent the location of ASFV-positive wild boars, with greenish ellipses representing their spatial cluster.

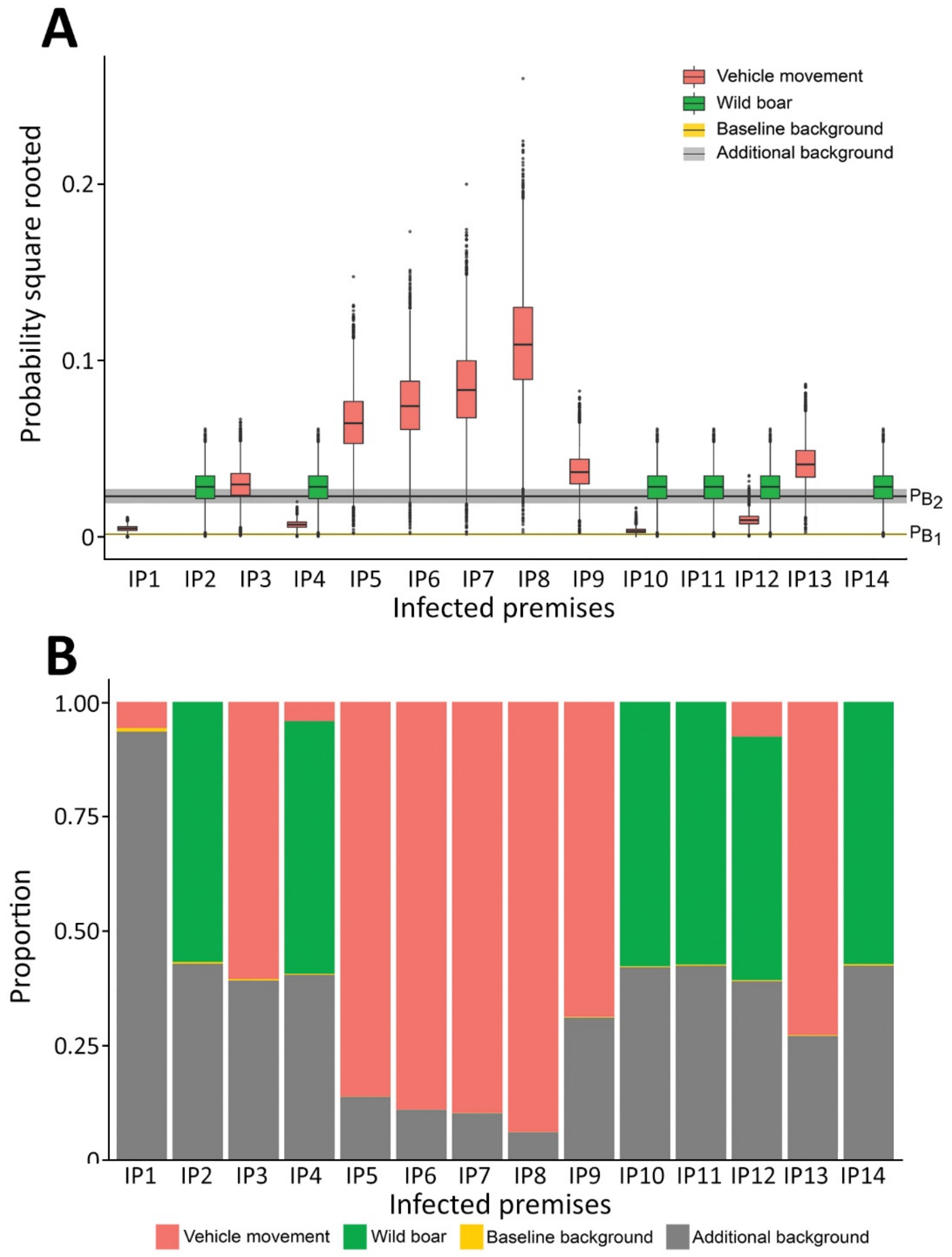

Appendix Figure 7. The force of infection on IPs via different transmission results (A) The force of infection exerted on IPs on the estimated dates of infection via different transmission routes. In the boxplots, center lines represent medians, and box limits represent upper and lower quartiles. Upper and lower whiskers extend to the largest and smallest values within 1.5x interquartile ranges, respectively. Points represent outliers. (B) The proportion of simulated infections caused by different transmission routes. ‘Baseline background’ represents a background risk for all farms in the country, and ‘additional background’ an additional background risk for farms in the epidemic region. The results are based on the MCMC output from the full model, with the three-day assumption on the duration of vehicle infectiousness.

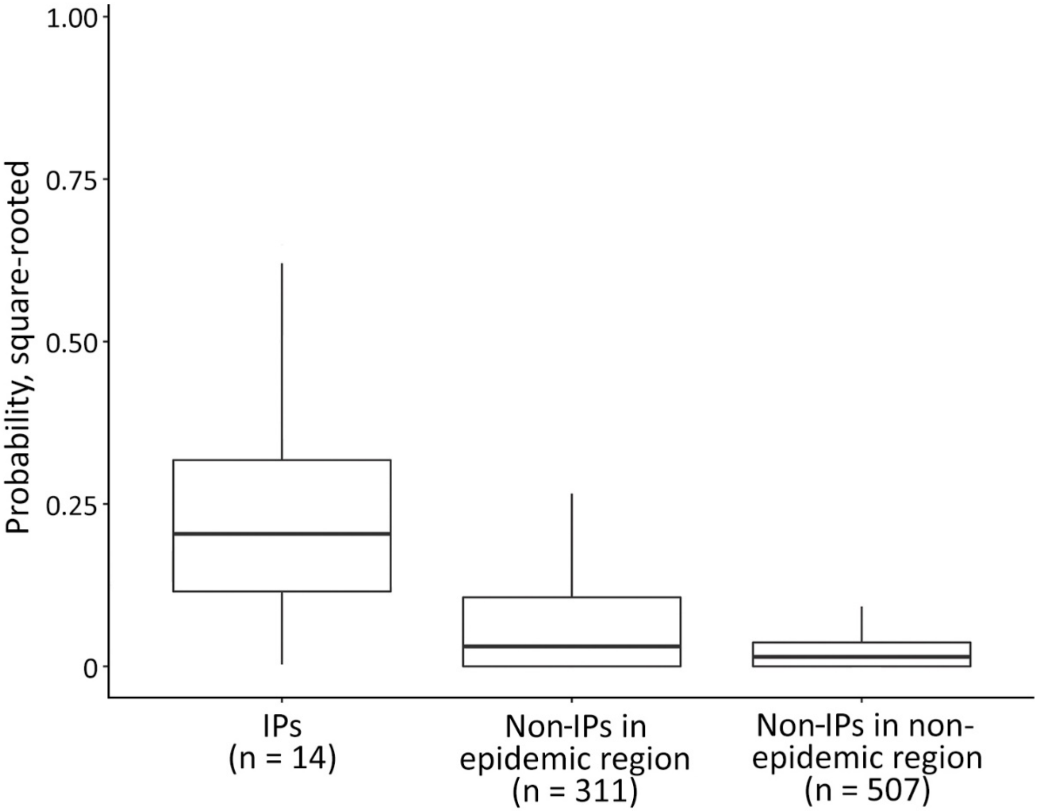

Appendix Figure 8. The force of infection resulting from potentially contaminating vehicle movements, based on the MCMC output from the full model. The comparison was made between IPs, non-IPs within and outside affected municipalities (Ganghwa island, Gimpo, Paju, and Yeoncheon), among farms visited by vehicles that visited IPs within three days, the assumed length of the vehicle infectious period. The number in each group (in parentheses) corresponds to the number of pig farms visited by vehicles that visited IPs at least once during the study period. In the boxplots, center lines represent medians. Box limits represent upper and lower quartiles. Upper and lower whiskers extend to the largest and smallest values within 1.5x interquartile ranges, respectively.

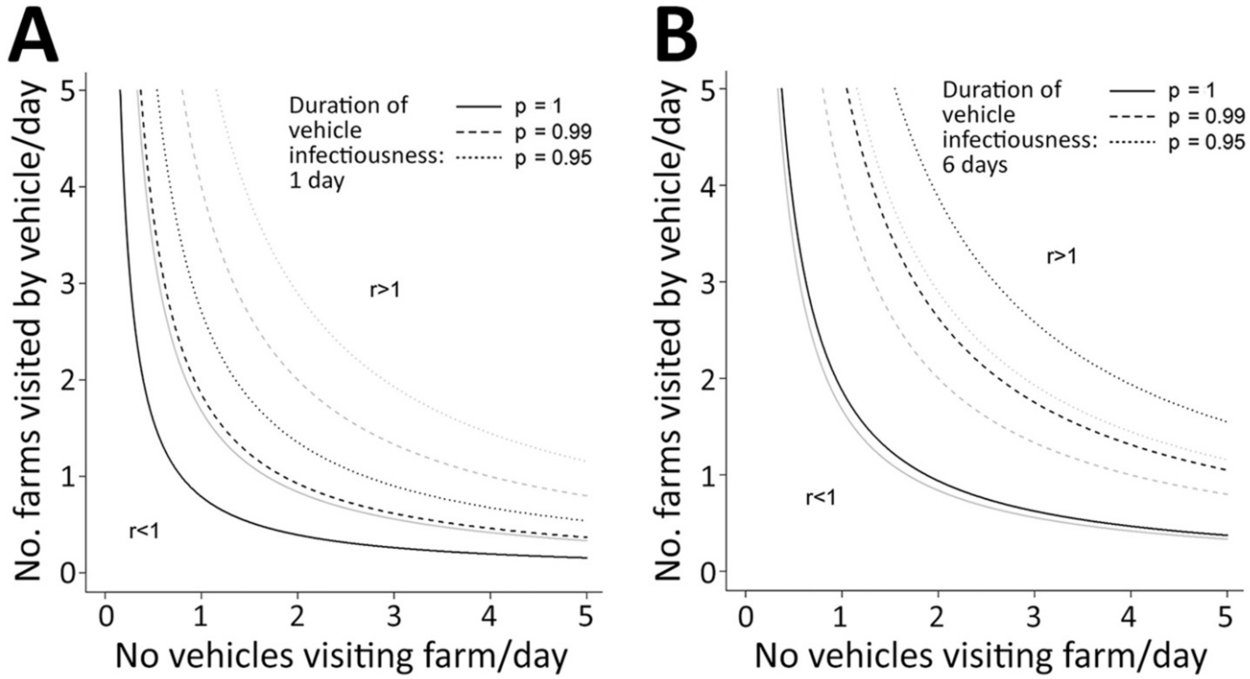

Appendix Figure 9. The expected number of secondary farm cases (r) caused by one infected farm through the movements of vehicles. r is computed as a function of the average daily number of vehicles visiting a farm (x-axis) and the average daily number of farms visited by a vehicle (y-axis). (a) and (b) are based on the one- and six-day assumptions on the duration of vehicle infectiousness. Different lines represent different thresholds for the proportion of iterations in which r was lower than one (p=1, 0.99, or 0.95). Pale grey lines represent the results when vehicles were assumed to remain infectious for three days after leaving an infected farm.

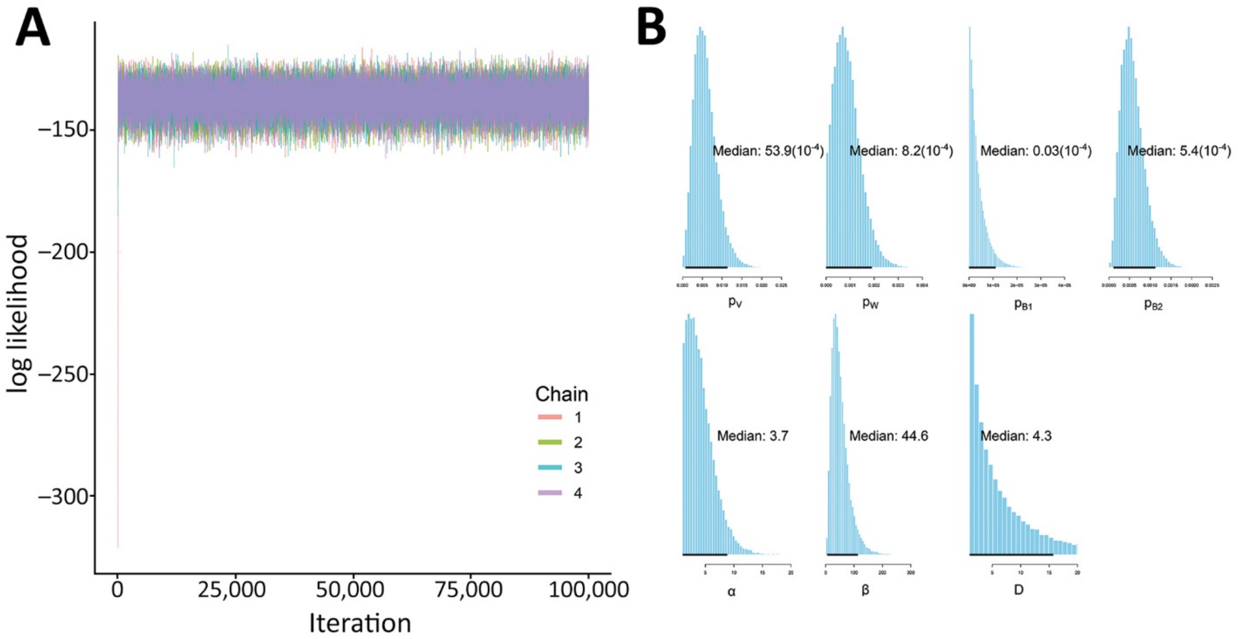

Appendix Figure 10. The MCMC outputs from the full model testing the contribution of both vehicle movements and wild boars in the spread of ASFV to domestic pig farms, with The three-day assumption on the duration of vehicle infectiousness. (A) The log-likelihood trace plot. Different colors represent chains with different starting values. (B) The posterior distributions of transmission (P_V, P_W, and P_B) and Gamma distribution (α and β) parameters, and the number of days between infection and reporting (D) (in days). The posterior distribution of D was simulated from the Gamma distribution with joint posterior α and β values as mean and variance. Thick black horizontal lines represent 95% highest-density intervals (HDIs).

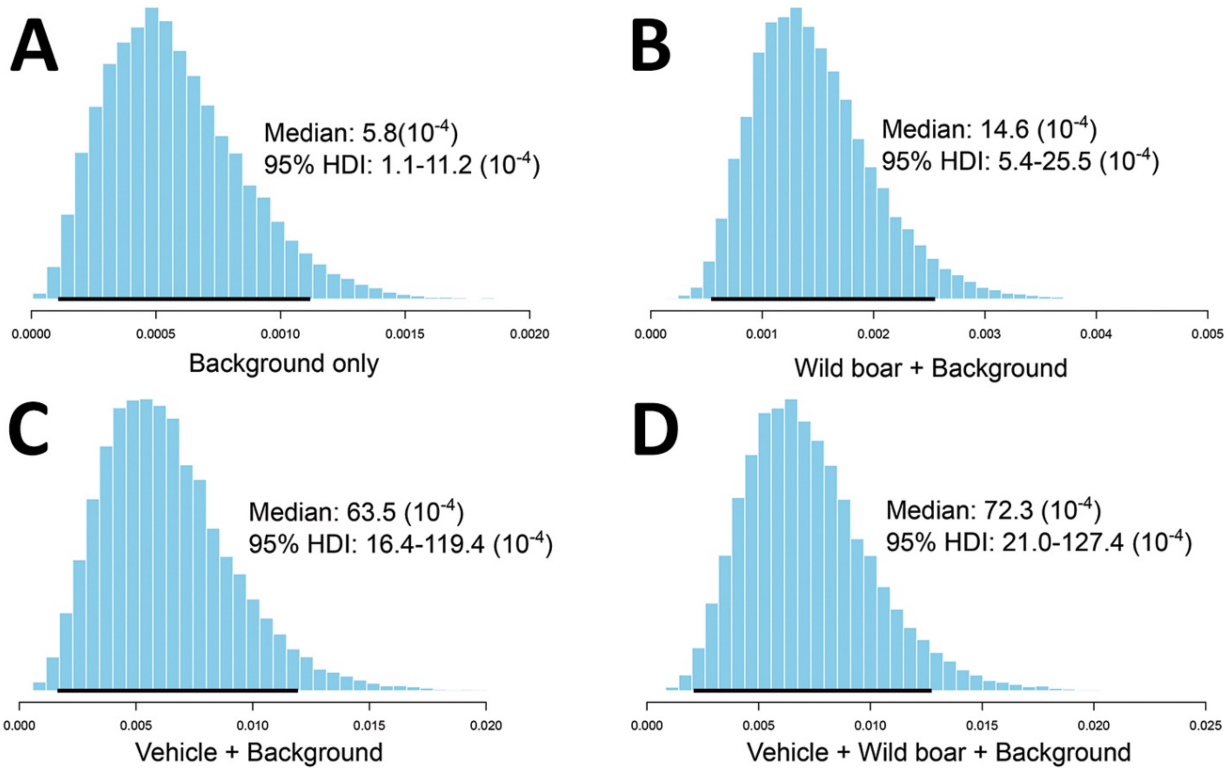

Appendix Figure 11. The daily force of infection for farms exposed to different combinations of the following transmission routes: (i) when visited by a single contaminated vehicle (‘Vehicle’), (ii) when located in a spatial cluster of ASFV-positive wild boars (‘Wild boar’), and (iii) when exposed to transmission routes other than contaminated livestock vehicles and infected wild boars (‘Baseline’). The results are based on the MCMC output from the full model, with the three-day assumption on the duration of vehicle infectiousness.
